# Some Gynæcological Cases

**Published:** 1885-02

**Authors:** F. R. Campbell


### Clinical Reports.

### Some Gynæcological Cases.

By F. R. Campbell, M. D.

*Case* 1. Removal of uterine fibroid. Mrs. G., age 34, multipara, has been troubled with dysmenorrhœa for the past twelve years. Five years ago the pain at the menstrual epochs was greatly increased in severity, and has been gradually growing more intense. There has been excessive menorrhagia for some time past, and the patient is now extremely anæmic, and somewhat jaundiced. Patient was first seen by me October 2, 1884. She was suffering the most agonizing pain, and flowing excessively. On making a digital examination I found the os dilated, and a tumor the size of a goose egg could be distinctly felt within the uterine cavity. The patient's temperature was at this time 104°, and, as she gave a history of intermittent fever, I prescribed quinine, and also morphine in sufficient quantities to relieve pain.

October 3d. Dr. C. C. Frederick was called in consultation. We examined patient under ether, and found that the tumor was a fibroid, sessile, and attached to the fundus of the uterus. It was decided to operate, as the patient was very anxious to be rid of her burden, even though the chances of recovery were slight.

October 6th. Although the patient was so weak that she could not leave her bed, and her temperature was 102°, we attempted the removal of the tumor by ecraseur. After several futile attempts, the wire breaking each time, we abandoned this method of operation. The cervix was incised bilaterally, a curved uterine scissors introduced and the tumor enucleated.

The cavity of the uterus was washed out with a solution of bichloride of mercury in warm water. There was but little hemorrhage, and the patient soon rallied from the shock and effects of the anæsthetic. The uterine injections were continued for two weeks, when all symptoms of pelvic inflammation disappeared. For four weeks, however, the fever, which had assumed a remittent type, continued and baffled all remedies. Quinine, gr. xx per diem, was finally given hypodermically with good results. The temperature gradually fell, but did not become normal.

Four weeks after the operation, a tumefaction appeared in the right hypochondriac region, causing considerable pain. In a few days there were evacuations of pus per rectum, and the swelling disappeared, with an improvement of the general condition of patient. A supporting treatment was continued with antipyrin, gr. x *bis die*, as the frequent hypodermic injections of quinine had irritated the skin. The patient has continued to improve, and writes me, Dec. 28th, that she is quite well, and has menstruated without pain for the first time in six years.

*Case* 2. Salivation by intra-uterine injections of bichloride of mercury. Mrs. W., age 18, had a miscarriage Oct. 17th, during the fourth month of utero-gestation. For ten days succeeding this event, she had frequent pains and hemorrhages, which became so alarming Oct. 27th that a physician was summoned. On examination, I found that the placenta was retained, but was unable to remove it, and consequently obliged to tampon the vagina. The patient's temperature was at this time 105° with all the symptoms of septicæmia.

In the morning, Oct. 28th, Dr. D. McNeil was called in, and we succeeded in removing the placenta piece by piece. Intra-uterine injections of solution of corrosive sublimate, 1 part to 1,000, were employed every ten hours, an antipyretic dose of quinine was given, and stimulants copiously administered. The temperature fell to 103° during the day, and the injections were given at longer intervals. Oct. 31st, the patient complained of a sore mouth and said she could not masticate food. The breath showed a distinct mercurial odor and the teeth subsequently became loose. The bichloride was stopped and liq. sodæ chlorinatis substituted. The patient continued to improve and eventually recovered after suffering a month with a severe attack of phlegmasia dolens.

*Case* 3. Muriate of cocaine in the treatment of venereal warts. Kate H. has had venereal warts for three years. The labia majora, the perinæum and even the inner aspect of the thighs were covered with these excrescences. Chromic acid was applied under anæsthesia in August last, but the operation only caused a great deal of suffering and the warts soon returned. Glacial acetic acid was applied to them in my office, but caused so much pain that the application could not be repeated.

I decided to try the new local anæsthetic in the treatment. A four per cent. solution was painted over the warts, which were afterward carefully saturated with glacial acetic acid. There was scarcely any pain in this method of treatment. The warts rapidly disappeared, and although two months have elapsed since the last application of the acid, there is no indication of their return. Fluid extract of thuja occidentalis was administered internally in connection with the local treatment.

*Case* 4. Removal of urethral caruncle, using muriate of cocaine as the anæsthetic. Mrs. B., age 29, has suffered for two years past from painful micturition and dyspanuria. After taking various proprietary remedies, including twenty-four bottles of "Safe Kidney Cure," she decided to consult a physician. On inspection, I found a urethral caruncle nearly as large as a chestnut, projecting from the urethra. Passing a probe along side of it, the pedicle was ascertained to be about half an inch in length. The tumor was so extremely sensitive that manipulation with the forceps was impossible. Absorbent cotton on a probe was saturated with a four per cent. solution of muriate of cocaine and applied for a few minutes to the caruncle. It could then be grasped with the forceps, and was removed with a Jarvis snare. There was but little pain experienced, except at the last, when the wire caused traction of the urethral mucous membrane, which had not been anæsthetized by the solution.

